# Influencing factors of intrapartum fever after epidural labor analgesia

**DOI:** 10.1590/1806-9282.20240565

**Published:** 2024-10-25

**Authors:** Xiaohua Yuan, Lin Qiu, Yuan Huang, Liyan Qu, Ping Zhu, Yingzi Zhang, Yonghui Yuan

**Affiliations:** 1 Fuzhou Maternal and Child Health Hospital, Department of Anesthesiology – Fuzhou, China.; 2 Fuzhou Maternal and Child Health Hospital, Department of Obstetrics – Fuzhou, China.

**Keywords:** Fever, Epidural anesthesia, Delivery, Parturient

## Abstract

**OBJECTIVE:**

The objective of this study was to investigate factors influencing intrapartum fever in parturients receiving epidural labor analgesia.

**METHODS:**

This study included 410 parturients who received epidural labor analgesia at the authors’ hospital between February 2022 and February 2024. Participants were divided into a fever group (>37.5℃) and a control group (<37.5℃) based on their body temperature post-analgesia. General data, gestational comorbidities, and intrapartum-related conditions were compared. Influencing factors were analyzed using the chi-squared test and logistic regression.

**RESULTS:**

Intrapartum fever occurred in 90 parturients (22.0%). Univariate analysis indicated that maternal age (p=0.046), parity (p=0.042), oxytocin use (p=0.041), and timing of analgesia (p<0.001) were associated with intrapartum fever. Multivariate analysis revealed that the timing of analgesia (OR 3.612, 95%CI 1.533–8.510) and amniotic fluid contamination degrees I (OR 1.072, 95%CI 1.012–3.082) and II (OR 2.874, 95%CI 1.901–9.092) were independent risk factors. No significant differences were found between the fever and control groups in body mass index, gestational age, gestational comorbidities, and artificial membrane rupture (p>0.05). Intrapartum fever increased the rate of neonatal fever within 2 h after birth (41.7 vs 18.6%, p<0.05) but did not significantly affect other neonatal health indicators.

**CONCLUSION:**

Timing of analgesia and amniotic fluid contamination are significant factors influencing intrapartum fever in parturients receiving epidural labor analgesia.

## INTRODUCTION

Childbirth is a necessary physiological process for parturients, and during normal delivery and cesarean section, labor pain will occur. With the advancements in anesthesia, more primiparas tend to use analgesia to reduce labor pain and improve delivery outcomes. The modes of spinal block for labor analgesia include epidural and subarachnoid block, with epidural block being more commonly used^1,2,3^. Epidural labor analgesia can effectively reduce pain and improve the rate of spontaneous delivery. In 1989, the Lancet first reported an association between epidural labor analgesia and intrapartum fever and found a significantly increased risk of fever in parturients receiving epidural analgesia at delivery^4^. Current studies have shown that the intrapartum febrile rate of Chinese parturients after receiving labor analgesia is between 10 and 35%^5,6^. Intrapartum pyrexia can trigger uterine inertia and prolong labor, thereby increasing the incidence of abnormal delivery and endometritis^7^. It may also lead to decreased fetal oxygen supply, fetal distress, meconium aspiration syndrome, and hypoxic-ischemic encephalopathy risk^8,9^.

Current research has identified various influencing factors for intrapartum fever, including maternal age, parity, oxytocin use, analgesia timing, and amniotic fluid contamination. Some studies suggest that early administration of labor analgesia may be associated with a higher incidence of fever due to prolonged labor and increased medical interventions^10^. Other factors, such as the number of vaginal examinations and the presence of infection, have also been linked to intrapartum fever^11^. However, the exact mechanisms and interactions between these factors remain inconclusive, necessitating further investigation. In this study, 410 parturients were used as the study participants to analyze the influencing factors of intrapartum fever in parturients who received epidural labor analgesia.

## METHODS

### Study participants

A total of 410 parturients hospitalized for delivery in the authors’ hospital between February 2022 and February 2024 were selected as the study participants and divided into a fever group and a control group according to whether fever occurred during labor following epidural labor analgesia. Fever was defined as a tympanic temperature of 37.5℃ or higher during labor. This threshold is consistent with the clinical definitions of intrapartum fever used in obstetric practice and research. For instance, Wang et al.^9^ and Zhao et al.^10^ employed similar criteria in their studies on intrapartum fever associated with epidural labor analgesia. The inclusion criteria were as follows: (1) women aged 18–42 years (this age range was chosen based on typical reproductive age guidelines and the increased risks associated with advanced maternal age; women over 42 years may have a higher baseline risk for complications, which could confound the study results); (2) no contraindications to epidural anesthesia and no history of allergy to anesthetic drugs; (3) voluntary epidural labor analgesia was received and signed informed consent was given by the patient and their families; (4) singleton, cephalic presentation; and (5) vaginal delivery by parturients. The exclusion criteria included: (1) body temperature >37.5℃ before labor analgesia and (2) incomplete clinical data. This study was approved by the ethics committee of the hospital. Parturients and their families signed informed consent forms.

### Study methods

The parturients who voluntarily received epidural labor analgesia were comprehensively and systematically assessed by the anesthesiologist, who opened the venous access, connected the electrocardiogram monitor, monitored blood pressure, pulse, and oxygen saturation, and continuously monitored the fetal heart rate. After a successful puncture at the L2–3 or L3–4 space, an epidural catheter connected to an analgesic pump was placed, and 12.5% ropivacaine combined with 0.5 μg/ral sufentanil plus normal saline to 120 mL (background volume 6–8 mL/h and maintenance volume 8 mL/h) patient-controlled analgesia (8 mL/h once with a time lock of 20 min) was administered until the end of delivery to maintain a relatively constant surrounding environment. Adequate energy intake was also ensured during analgesia.

According to the anesthetic effect, motor block, and complications, the anesthesiologists could adjust the anesthesia at any time. When the parturients experienced fulminant pain, the anesthesiologists performed a timely check of the position of the epidural catheter and the administration of the analgesic pump to determine the dose and the concentration of the analgesia administration according to the characteristics of the pain, the general condition of the parturients and fetuses, the progress of labor, and maternal need. If necessary, appropriate concentrations of anesthetic drugs would be added, and the vital signs of parturients were closely observed until at least 30 min after the additional drugs were administered. If there was no significant improvement, superior physicians would be asked to consider re-puncture and catheterization. If the parturients experienced decreased blood pressure, it was determined whether it was supine hypotension. Assisting parturients into the lateral decubitus position, oxygen inhalation, medication, and other methods were used to increase blood pressure. If there was itching and erythema, timely intravenous use of calcium gluconate or dexamethasone and other anti-allergic drugs was made. In the case of nausea or vomiting, rule out the cause of hypotension, antiemetics could be appropriately applied. If the parturient had urinary retention, the bladder filling degree was checked, and temporary catheterization could be given. If the fetal heart rate was abnormal, the obstetrician conducted a timely analysis and evaluation and performed a cesarean section if necessary. After the end of delivery, a determination that the parturient had no abnormalities was made, and the epidural catheter was removed.

### Outcome indicators

The general data of the parturients was collected, including age, body mass index (BMI), gestational age, parity, and gestational comorbidities (e.g., gestational diabetes mellitus and gestational anemia). The maternal intrapartum-related conditions were noted as follows: oxytocin requirements, artificial membrane rupture, amniotic fluid pollution degree, and the timing of the analgesia. The general health status of the newborns was recorded: Apgar score at 1–5 min, presence or absence of fever within 2 h after birth, transcutaneous bilirubin value, use of antibiotics, and length of hospital stay. Data related to the condition of the newborns were collected immediately after birth and within the first 2 h postpartum to assess the immediate effects of maternal intrapartum fever.

### Statistical analysis

Statistical analysis of the data was performed using IBM SPSS 26.0. Continuous variables were analyzed using the t-test, categorical variables were analyzed using the chi-squared (χ^2^) test, and multivariate logistic regression analysis was used to determine the influencing factors of intrapartum fever after epidural labor analgesia. A p-value of <0.05 was considered statistically significant.

## RESULTS

### Univariate analysis of influencing factors of intrapartum fever

There were no significant differences between the fever and control groups in terms of BMI, gestational age, and gestational comorbidities (e.g., gestational diabetes mellitus and gestational anemia). A total of 410 parturients with a mean age of 28.5 years receiving epidural labor analgesia were included in this study. There were 278 primiparous women (67.8%) and 18 multiparous women (4.4%) who had delivered twice or more. Ninety parturients developed intrapartum fever, accounting for 22.0%. The results showed that there were significant differences between the fever and afebrile groups in terms of age (p=0.046) and parity (p=0.042), whether oxytocin was used during the delivery (p=0.041) and the timing of the analgesia (p<0.001) (p<0.05) (Table 1).

**Table 1:** Single-factor analysis of influencing factors of intrapartum fever.

Item	Control group	Fever group	χ^2^ value	p-value
Age (year)	28.05±4.23	29.19±2.25	4.05	0.046
BMI (Kg/m^2^)	27.35±4.02	28.86±4.72	2.155	0.142
Parity				
Primiparous	212	66	5.448	0.042
Parous one	97	17		
Delivered twice or more	11	7		
Gestational week (weeks)				
≤37	6	1	1.225	0.268
>37	314	89		
Disease conditions				
Gestational diabetes				
No	245	66	0.195	0.654
Yes	75	24		
Anemia of pregnancy				
No	215	57	0.035	0.844
Yes	105	33		
Oxytocin use				
No	117	21	4.12	0.041
Yes	203	69		
Analgesic timing				
2–3 cm	219	81	17.115	<0.001
≥3 cm	101	9		
Artificial rupture of the embryo membrane				
No	91	22	0.521	0.755
Yes	162	47		
PROM	67	21		
Amniotic fluid cleanliness			5.525	0.228
Amniotic fluid clean	264	65		
Contamination degree I	9	5		
Contamination degree II	13	8		
Contamination degree III	33	12		
Bloody amniotic fluid	1	0		

BMI: body mass index; PROM: premature rupture of membranes.

### Multivariate analysis of influencing factors of intrapartum fever

After determining the absence of collinearity between the variables, independent variables were included in the multivariate logistic regression analysis with the presence or absence of intrapartum fever following labor analgesia as the dependent variable (fever=1, no fever=0). Multivariate logistic regression analysis showed that the premature timing of analgesia and amniotic fluid contamination were independent risk factors affecting intrapartum fever in parturients (Table 2).

**Table 2:** Logistic regression analysis of influencing factors of fever.

Item	p-value	OR value	95%CI
Age (years)			
<25		1	
25–29	0.785	0.902	0.415–1.942
29–34	0.102	0.44	0.162–1.750
≥35	0.415	0.58	0.155–2.125
BMI (kg/m^2^)			
Normal		1	
Overweight	0.094	2.284	0.858–6.057
Obese	0.092	2.281	0.864–5.995
Parity			
Primiparous		1	
Parous one	0.312	0.661	0.294–1.485
Delivered twice or more	0.255	2.581	0.491–12.485
Gestational week (weeks)			
≤37		1	
>37	-	-	-
Disease conditions			
Gestational diabetes			
No		1	
Yes	0.812	0.925	0.502–1.712
Anemia of pregnancy			
No		1	
Yes	0.432	0.798	0.450–1.402
Oxytocin use			
No		1	
Yes	0.442	1.302	0.661–2.566
Analgesic timing			
2–3 cm	0.003	3.612	1.533–8.510
≥3 cm		1	
Artificial rupture of the embryo membrane			
No		1	
Yes	0.601	1.235	0.552–2.761
PROM	0.581	1.21	0.607–2.410
Amniotic fluid cleanliness			
Amniotic fluid clean		1	
Contamination degree I	0.041	1.072	1.012–3.082
Contamination degree II	0.019	2.874	1.901–9.092
Contamination degree III	0.171	1.86	0.742–4.655
Bloody amniotic fluid	-	-	-

BMI: body mass index; CI: confidence interval.

### Analysis of the effect of intrapartum fever on the general health status of newborns

The condition of newborns was assessed based on several indicators. The results showed that intrapartum fever in parturients was associated with an increased rate of fever in newborns within 2 h after birth (41.7% in newborns of parturients with intrapartum fever vs. 18.6% in newborns of parturients without intrapartum fever, p<0.001). However, there were no significant differences in other neonatal indicators between the two groups, such as the length of hospital stay, antibiotic use, Apgar score at 1–5 min, and transcutaneous bilirubin values (p>0.05) (Table 3).

**Table 3:** Analysis of the effect of intrapartum fever on the general health status of newborns.

Item	Total number	Fever detection number	χ^2^ value	p-value
Neonatal hospital stay (days)				
≤3	303	63	0.351	0.552
>3	107	27		
Antibiotic use in neonates				
No	332	70	0.585	0.441
Yes	78	20		
Apgar score at 1 min				
8	12	6	1.755	0.752
9	14	9		
10	384	75		
Apgar score at 5 min				
8	8	1	4.116	0.218
9	16	14		
10	386	75		
Neonatal jaundice index				
Normal	252	48	2.01	0.151
High	158	42		
Neonatal fever 2 h				
No	350	65	11.355	0.001
Yes	60	25		

## DISCUSSION

In this study, the factors that influenced intrapartum fever in parturients receiving epidural labor analgesia were maternal age, parity, oxytocin use during delivery, and the timing of labor analgesia. Multivariate logistic regression analysis showed that a high number of gynecological internal examinations, the early timing of labor analgesia, and amniotic fluid contamination were independent risk factors for intrapartum fever following epidural labor analgesia. The secondary conclusion was that intrapartum fever increased the rate of fever in neonates within 2 h after birth but had no effect on the length of the neonatal hospital stay, use of antibiotics, Apgar score at 1–5 min, and transcutaneous bilirubin values.

A total of 410 parturients receiving epidural labor analgesia were included in this study, with a mean age of 28.5 years. There were 278 primiparous women (67.8%) and 18 multiparous women (4.4%) who had delivered twice or more. Ninety parturients developed intrapartum fever, accounting for 22.0%, which was consistent with previous findings^8,12^. Labor analgesia is associated with intrapartum fever, which may be due to reduced heat dissipation by blocking the sympathetic nerves and reducing sweat gland secretion^13^. Anesthetic drugs may activate the immune system, producing relevant inflammatory factors and triggering an aseptic inflammatory response^14^. Labor analgesia may prolong labor and increase uncertainty factors that lead to intrapartum fever while waiting for delivery^15^. Previous studies have shown that the number of gynecological examinations is an independent risk factor for intrapartum fever in parturients, possibly because the probability of vaginal mucosal damage increases with the number of gynecological examinations, which, in turn, increases the risk of infection that triggers intrapartum fever^16,17^. It is suggested that medical staff minimize the number of vaginal examinations during maternal delivery and strictly implement aseptic techniques during the examinations. In addition, amniotic fluid contamination is also one of the risk factors for the emergence of maternal intrapartum fevers^18^. After the appearance of amniotic fluid contamination, the possibility of maternal infection is greatly increased, which may lead to the occurrence of intrapartum fever. Our findings align with these results, showing that amniotic fluid contamination significantly increases the risk of intrapartum fever (odds ratio=1.860, 95%CI 0.742–4.655, p=0.042). This is consistent with the literature, which suggests that contaminated amniotic fluid can increase the risk of infection and inflammation, thereby contributing to maternal fever during labor.

In this study, we found that the earlier the timing of analgesia, the higher the likelihood of intrapartum fever, consistent with certain studies^19^. The reason for this study’s finding may be that the earlier the timing of analgesia, the lower the hormone content of the uterine smooth muscle contractions, which leads to weakened contractions and prolonged labor and thus increases the risk of intrapartum fever^20^. It has been shown that the artificial rupture of the membranes during delivery increases the intrapartum fever rate^21^, although the results of this study showed no significant difference. One reason for this may be that the majority of the selected subjects had premature rupture of the membranes, resulting in a small number of samples of the artificial rupture of the membranes and a lack of an adequate number of participants, thus reducing the power of the test. The other should strictly adhere to the indications for artificial rupture of membranes and meticulously follow operating standards and aseptic principles. By doing so, they can likely avoid the risk of infection associated with obstetric interventions.

In this study, we also found that fever following epidural labor analgesia in parturients caused an increase in neonatal body temperature within 2 h after birth. One possible reason may be that the fetus avoids hyperthermia by delivering heat to the mother, meaning maternal fever leads to the obstruction of fetal heat dissipation and thus increases fetal temperature, resulting in the inability of the neonate’s temperature to drop to normal for a short time after birth^22^. However, there was no significant difference in other neonatal indicators, including the neonatal length of stay in hospital, use of antibiotics, Apgar score at 1–5 min, and transcutaneous bilirubin values. The reasons for this may be: (1) insufficient sample size, resulting in reduced power of testing; and (2) that epidural-related intrapartum fever is mostly transient with no other discomfort symptoms, and certain interventions are often taken for this in clinical practice (e.g., the use of antibiotics, physical cooling, and accelerating labor), meaning adverse neonatal outcomes may be related to the medical interventions taken rather than directly related to epidural-related intrapartum fever.

This study’s comprehensive analysis of multiple potential risk factors for intrapartum fever, including maternal age, parity, oxytocin use, timing of analgesia, and amniotic fluid contamination, is a key highlight, providing robust data with a large sample size of 410 parturients. However, the study’s retrospective design may introduce selection bias and limit the ability to establish causal relationships. As a single-center study, the findings may not be generalizable to other settings or populations with different demographics and clinical practices. The scope of neonatal outcomes assessed is limited to immediate effects, without long-term follow-up, and potential confounding variables that were not measured may influence the results. Future studies should address these limitations and explore additional factors influencing intrapartum fever.

## CONCLUSION

In summary, this study identified early timing of analgesia and amniotic fluid contamination as significant independent risk factors for intrapartum fever in parturients receiving epidural labor analgesia. Maternal intrapartum fever was associated with an increased rate of neonatal fever within the first 2 h postpartum but did not significantly impact other neonatal outcomes. Effective management of labor analgesia timing and monitoring amniotic fluid can help reduce the incidence of intrapartum fever and improve maternal and neonatal outcomes. Future research should include multicenter studies and long-term follow-up to better understand the broader implications of intrapartum fever.

## References

[1] Khairudin MN, Vallikkannu N, Gan F, Hamdan M, Tan PC (2024). Electric massage chairs reduce labor pain in nulliparous patients: a randomized crossover trial. Am J Obstet Gynecol MFM.

[2] Jia C, Zou B, Sun YJ, Han B, Diao YG, Li YT (2024). The 90% effective concentration of alfentanil combined with 0.075% ropivacaine for epidural labor analgesia: a single-center, prospective, double-blind sequential allocation biased-coin design. J Anesth.

[3] Cai L, Jiang JJ, Wang TT, Cao S (2023). Effects of combined spinal-epidural anesthesia on anxiety, labor analgesia and motor blocks in women during natural delivery. World J Psychiatry.

[4] Fusi L, Steer PJ, Maresh MJ, Beard RW (1989). Maternal pyrexia associated with the use of epidural analgesia in labour. Lancet.

[5] Li H, Yang L, Peng J, Cheng W, Ma H, Wu S (2024). Duration time of labor progression for pregnant women of vaginal birth after cesarean in Hubei, China. Ir J Med Sci.

[6] Chen Z, Zhu C, Huang L, Qi Y, Guo X, Xie L (2023). Serum magnesium level as a biomarker to predict the risk of labor epidural anesthesia associated fever. Int J Gen Med.

[7] Yuan J, Jin A, Shen J, Chen Y, Huang Q, Xiang H (2024). Maternal intrapartum fever during epidural labour analgesia: incidence and influencing factors. Int J Nurs Pract.

[8] Zhang Z, Deng CM, Ma JH, Li S, Lei B, Ding T (2023). Effects of neuraxial labor analgesia on intrapartum maternal fever in full-term pregnancy and its influence on birth outcomes. Front Med (Lausanne).

[9] Wang H, Yang Z, Wei S, Xia L, Li Y, Wu X (2023). Perinatal outcomes and risk factors for epidural analgesia-associated intrapartum maternal fever: a retrospective study. J Matern Fetal Neonatal Med.

[10] Zhao B, Li B, Wang Q, Song X (2022). The relationship between epidural analgesia and intrapartum maternal fever and the consequences for maternal and neonatal outcomes: a prospective observational study. J Matern Fetal Neonatal Med.

[11] Sultan P, Segal S (2020). Epidural-related maternal fever: still a hot topic, but what are the burning issues?. Anesth Analg.

[12] Yao Z, Zhou J, Li S, Zhou W (2022). The effects of combined spinal-epidural analgesia and epidural anesthesia on maternal intrapartum temperature: a randomized controlled trial. BMC Anesthesiol.

[13] Chu Q, Sun Y, Bai L, Bai Y, Zhang D, Zheng P (2022). Combined spinal-epidural analgesia and epidural analgesia induced maternal fever with a similar timing during labor-A randomized controlled clinical trial. Front Med (Lausanne).

[14] Chen MS, Hu CL, Jiang SK, Chong ZY, Chen JC (2024). Modulation of secretory factors by lipofundin contributes to its anti-neuroinflammatory effects. Exp Ther Med.

[15] Sun C, Ren S, Chen C, Liu Q, Lu P, Xia L (2022). Pulse perfusion index for predicting intrapartum fever during epidural analgesia. J Clin Anesth.

[16] Wohlrab P, Boehme S, Kaun C, Wojta J, Spittler A, Saleh L (2020). Ropivacaine activates multiple proapoptotic and inflammatory signaling pathways that might subsume to trigger epidural-related maternal fever. Anesth Analg.

[17] Yin H, Hu R (2022). Risk factors of maternal intrapartum fever and the effect of fever duration on neonatal morbidity in different temperature. J Obstet Gynaecol Res.

[18] Jung E, Romero R, Suksai M, Gotsch F, Chaemsaithong P, Erez O (2024). Clinical chorioamnionitis at term: definition, pathogenesis, microbiology, diagnosis, and treatment. Am J Obstet Gynecol.

[19] Li B, Liao Y, Wang Q, He S, Yang L, Jia J (2023). Association between epidural-related maternal fever and short-and long-term prognosis of parturients: a prospective observational study. Front Surg.

[20] Yan M, Wang Q, Zhang Y, Zhou J, Cui E, Sun J (2023). Application of dural puncture epidural technique combined with programmed intermittent epidural bolus mode in labor analgesia: a randomized clinical trial. Medicine (Baltimore).

[21] Callahan EC, Lee W, Aleshi P, George RB (2023). Modern labor epidural analgesia: implications for labor outcomes and maternal-fetal health. Am J Obstet Gynecol.

[22] Qu L, Yang Y, Yin Y, Yin TT, Zhang X, Zhou X (2023). Analysis of the maternal and fetal adverse outcomes of 154 pregnant women with cesarean section in the second stage of labor. Zhonghua Fu Chan Ke Za Zhi.

